# Development of a vaccine against the synthetic opioid U-47700

**DOI:** 10.3389/fphar.2023.1219985

**Published:** 2023-07-10

**Authors:** Hyeri Park, Mingliang Lin, Jian Zhou, Lisa M. Eubanks, Bin Zhou, Kim D. Janda

**Affiliations:** ^1^ Departments of Chemistry and Immunology, The Skaggs Institute for Chemical Biology, Worm Institute for Research and Medicine (WIRM), The Scripps Research Institute, La Jolla, CA, United States; ^2^ The College of Chemistry, Nankai University, Tianjin, China

**Keywords:** new psychoactive substance, U-47700, overdose, conjugate vaccine, immunopharmacotherapy

## Abstract

Opioid use disorders and overdose have become a major public health concern in recent years. U-47700, a New psychoactive substances (NPS) opioid, also known as “pinky” or “pink” has been identified as a new threat in the drug supply because of its potency and abuse potential. Conjugate vaccines that can produce antibodies against target drug molecules have emerged as a promising tool to treat substance use disorders. Herein, we report the design, synthesis, and *in vivo* characterization of a U-47700 vaccine. The vaccine demonstrated favorable results with rodents producing elevated levels of antibody titer and sub-micromolar affinity to U-47700. In addition, antibodies generated by the vaccine effectively mitigated drug-induced effects by preventing the drug from penetrating the blood-brain barrier, which was verified by antinociception and drug biodistribution studies. The development of a vaccine against U-47700 and other NPS opioids contributes to the continued advancement of non-conventional pharmacological treatments to address the global opioid epidemic.

## 1 Introduction

The opioid epidemic has affected millions of lives worldwide and to compound matters worse an increase in opioid related overdoses have been linked to the COVID-19 pandemic (Friedman and Akre, 2021; Mattson et al., 2021). Moreover, there is growing evidence that new psychoactive substances (NPS) are fueling another arm in drug related overdose deaths that being added as unknown adulterants (Ghose et al., 2022). NPS is a group of synthetic drugs that are designed to produce enhanced psychoactive effects and more than 1150 NPS have been identified (UNODC, 2022).

**FIGURE 1:**
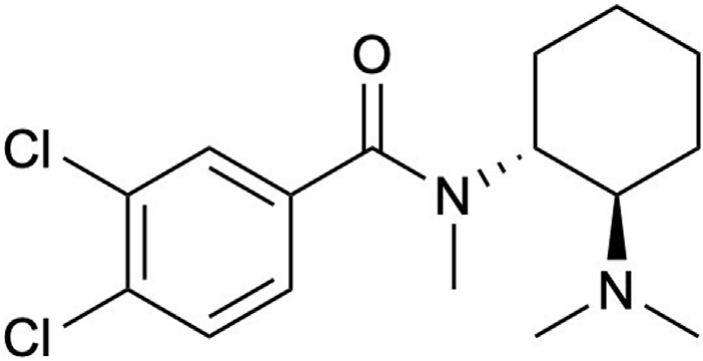
The structure of U-47700.

A prominent addition to this group is U-47700, 3,4-dichloro-*N*-((1*R*,2*R*)-2-(dimethylamino)cyclohexyl)-*N*-methyl benzamide; a synthetic opioid that was developed in the 1970s as a replacement for morphine (Szmuszkovicz and Kalamazoo, 1978; Szmuszkovicz and Von Voigtlander, 1982; Cheney et al., 1985). From a pharmacological perspective U-47700 also known as “pinky” or “pink” possesses as a similar chemical structure to other opioids like fentanyl (Figure 1), and binds to the *μ*-opioid receptors in the brain analogous to morphine inducing pharmacological effects such as pain relief, euphoria, and respiratory depression; yet it is also incredibly potent generating *in vivo* activities 7.5–12-fold greater than morphine (Kundu et al., 1999; Coopman et al., 2016; Domanski et al., 2017; Ellefsen et al., 2017). Furthermore, in the past decade, U-47700 has been associated with numerous deaths and overdoses worldwide, leading to additional public health risks. U-47700 combined with fentanyl and flubromazepam has been attributed to fatalities in Belgium, Germany, Ireland, and Italy. In 2016, at least 15 confirmed fatalities were reported, and by December 2017, the use of U-47700 was linked to a minimum of 46 deaths in US. (Mohr et al., 2016; Schneir et al., 2017; Koch et al., 2018; Krotulski et al., 2018; Moody et al., 2018).

Hand in glove with the opioid crisis is a need for new innovative approaches to prevent opioid drug related relapse and overdose. The traditional pharmacological treatments for substance use disorders aim to modulate the effects of a drug at the site(s) of actions, which is the pharmacodynamic strategy. Current treatments using this tactic for synthetic opioid substance use disorder and overdose have relied on such drugs as naloxone, naltrexone, methadone, and buprenorphine, which act as either antagonists, agonists or both upon the *μ*-opioid receptors (Ayanga et al., 2016; Volkow and Collins, 2017). While these traditional treatments for opioid addiction have been effective, they also possess side effects and pose a risk of medication abuse (Volkow et al., 2018). The challenges related to initiating and adhering to the treatment also demand significant attention and consideration. In view of these limitations immunopharmacotherapy was developed which is a pharmacokinetic approach engaging antibodies/vaccines that target the drug molecule itself (Gorelick, 2012; Bremer and Janda, 2017). Our laboratory has been one of the pioneers in developing synthetic opioid conjugate vaccines to combat drug addiction (Bremer et al., 2016; Raleigh et al., 2019; Barrientos et al., 2020; Stone et al., 2021). The general agenda of these drug conjugate vaccines includes a drug-like molecule attached to an immunogenic carrier protein, and an adjuvant. This “vaccine cocktail” when administered can stimulate the immune system producing antibodies against the selected drug. Moreover, these anti-drug antibodies limit the drug to the bloodstream, thus blunting blood brain penetration, which ultimately mitigates reward signaling or drug effects by reducing the interaction between the drug and central nervous system (CNS). In contrast to traditional pharmacodynamic treatments, the advantages of immunopharmacotherapy offer long-term protection and lack of abuse potential (Pravetoni and Comer, 2019).

In response to recent rising concerns about U-47700 as a NPS opioid, we have developed a vaccine for its prophylaxis. As the starting point, we designed a hapten to generate specific antibodies to target U-47700 molecules by considering the structural complexity of the drug. Thus, a drug hapten chimera was chemically synthesized and conjugated to an immunogenic carrier protein, keyhole limpet hemocyanin (KLH). The vaccine cocktail was formulated with two adjuvants, alum and CpG ODN 1826, to enhance the immune response. Immunization of the vaccine was conducted using Swiss Webster female mice and initially evaluated through examination of antibody titers and affinity. To access the vaccines protective properties against U-47700, antinociception and blood-brain biodistribution (BBB) studies were conducted. Using this as our research guide, we report our findings on a vaccine to attenuate U-47700 analgesic effects.

## 2 Materials and methods

### 2.1 Chemicals and drug

All chemicals and reagents were purchased from Aldrich and Combi-Block and used without further purification, unless otherwise stated. All solvents were American Chemical Society (ACS) grade or better and used without further purification. All reactions were conducted in oven-dried glassware under argon. Analytical thin layer chromatography (TLC) was performed with glass backed silica gel (0.25 mM thick, 60 Å) plates purchased from Sigma and visualized under UV irradiation at 254 nm and/or by staining with potassium permanganate solution followed by heating. Flash automated column chromatography was performed using a CombiFlash Rf + Luman (Teledyne Isco) purification system with flash silica RediSep Rf columns for normal phase (NP) or RediSep Rf Gold C_18_ HP columns for reverse phase (RP).

U-47700 was synthesized in-house following known procedures (McConnell et al., 2018). U-47700_d_6_ was purchased from Cayman Chemical Company.

### 2.2 Bioconjugation

An immunoconjugate of U-47700-KLH was prepared in parallel with bovine serum albumin (BSA) as a protein reference. The hapten in DMF was activated with 2-4 equivalents of 1-ethyl-3-(3-dimethylaminopropyl)carbodiimide (EDC) and *N*-hydroxysuccinimide (NHS) at room temperature overnight. The reaction was monitored by LCMS. After completing the reaction, the activated hapten was dried and reconstituted in DMSO. The protein solution (BSA or KLH, 1 mg/mL in PBS) was added to the activated hapten solution with a final DMSO concentration of less than 10%, and the coupling reaction was allowed to continue overnight at 4 °C using gentle end-over-end mixing. The reaction solution was dialyzed against pH 7.4 PBS buffer using a Slide-A-Lyzer 10 K MWCO dialysis cassette (Thermo Scientific) at rt. The buffer was exchanged every 2 h for 4 h, and then dialysis was continued overnight at 4°C. The conjugates were quantified by BCA assay (BCA™ protein kit, Thermo Scientific 23235). The hapten-BSA conjugate was analyzed by MALDI-TOF for the hapten:carrier protein conjugate number.

### 2.3 Animals

Eight-week-old female Swiss Webster mice (*n* = 6/group) were obtained from Taconic Farms (Germantown, NY) and allowed to acclimate for approximately 1 week before vaccination. Mice were group-housed in an AAALAC-accredited vivarium containing temperature- and humidity-controlled rooms, with mice kept on a reverse light cycle (light on: 9 p.m. to 9 a.m.). All animal studies were performed in compliance with the Scripps Institutional Animal Care and Use Committee and were in accordance with the National Institutes of Health Guild for the Care and Use of Laboratory Animals. All experiments were performed during the dark phase, generally between 1 p.m. and 5 p.m.

### 2.4 Vaccination

Each mouse received 200 μL of a vaccine formulated with 50 μg of KLH or U-47700-KLH, 50 μg of CpG ODN 1826 (Eurofins MWG Operon), and 500 μg of alum (Alhydrogel^®^, Invivogen) in PBS pH 7.4. Vaccines were made fresh and mixed for at least 1 h, prior to injection. The suspension (200 μL per mouse) was administered intraperitoneally on week 0, 2, and 4. No adverse reactions were observed, and all mice maintained a healthy weight throughout the vaccine study. Blood sampling was performed via retro-orbital sinus on week 3 and 5. Whole blood samples were centrifuged at 13,000 rpm for 15 min to separate the sera. All sera were stored at −20°C until further analysis.

### 2.5 Enzyme-linked immunosorbent assay (ELISA)

Microtiter plates (Corning^®^ 3690) were coated with 25 ng (1 μg/mL, 25 μL per well) of U-47700-BSA conjugate in PBS pH 7.4 overnight at 4°C. The plates were washed with dH_2_O before being blocked with 5% skim milk in PBS pH 7.4 by incubating at room temperature for 45 min. Blocking solution was removed, and mouse serum was serially diluted 1:1 in 1% BSA-PBS pH 7.4 across the plates starting at 1:200. The plates were incubated for 2 h at 37°C. After the incubation, the plates were washed with 10 times with dH_2_O, followed by incubation with horseradish peroxidase (HRP)-conjugated secondary antibody (donkey anti-mouse IgG, Jackson ImmunoResearch 715-035-151) diluted 1:10,000 in 1% BSA-PBS pH 7.4 for 1 h at 37°C. The plates were further washed 10 times with dH_2_O before being developed with 3,3′,5,5′-tetramethylbenzidine (TMB) substrate (Thermo Scientific). TMB substrate (40 μL) was incubated for 8–10 min at room temperature, and the reaction was quenched with 2 M aq. H_2_SO_4_ (40 μL). Plates were read with the absorbance at 450 nm. The absorbance values using GraphPad PRISM 8 were normalized to the highest absorbance value per set, and a curve was fit using the log(inhibitor) vs. normalized response–variable slope equation to determine midpoint titer and standard error.

### 2.6 Assessment of drug binding affinity

The IC_50_ values of U-47700 for immunized sera were determined by a competitive binding assay using surface plasmon resonance spectroscopy. Thus, a Biacore 3000 instrument (GE Healthcare Life Sciences) was engaged that was equipped with a research-grade CM5 sensor chip. The default protocol under application of “Wizard” in the Biacore control software was employed, and 1X HBS-EP + buffer was used as the running buffer. The ligand was immobilized in the flow cell (Fc) using an amine coupling methodology in the order as follows: 1) Fc1 with BSA as reference; 2) Fc2 with U-47700-BSA conjugate. Prior to immobilization, pH scouting was performed to evaluate the immobilization conditions. A 2% solution of 0.5 mg/mL BSA conjugate in pH 4.0 sodium acetate buffer (10 μg/mL) was used, and the immobilization was achieved through amide coupling via EDC/NHS activation followed by incubating with BSA conjugate at a flow rate of 5 μg/mL for 12 min, resulting in a 900RU signal increase.

Serum titration was determined by running serum samples at different dilutions through the coating Fc until the response was down to 100RU after the dissociation phase. A dilution of 1:40 K was selected, and the analyte was run at concentrations of 100 μM and 1 μM to test its affinity strength. A nearly complete inhibition was observed at 1 μM; thus, the range of inhibitor concentration was set from 10 μM to 10 nM (12 points). In the formal test, the diluted serum was pre-incubated with the analyte for 30 min at room temperature on a 600 rpm shaker. Each sample was injected for 5 min and dissociated for 2.5 min. The chip’s surface was then regenerated by injection of 10 mM Gly-HCl (pH 1.5) for 30 s before the next round of assay. Due to the strong carryover of the analyte, the assay was performed at a sequence of increasing inhibitor concentration, and a blank injection of 5% DMSO in HBS-EP + buffer was repeated five times before testing the next serum sample. The binding responses (Fc2-Fc1) for each sample were recorded in the sensorgrams, and the IC_50_ value for each compound was determined by plotting the normalized binding responses against compound concentrations. The IC_50_ values were further derived from a nonlinear fit of the binding curves using GraphPad Prism 8.

### 2.7 Antinociception

Nociception was measured in two behavior tests, hot plate (supraspinal) and tail flick (spinal), as previously described (Bremer et al., 2014). In the hot plate test, mice were placed in an acrylic cylinder (14 cm in diameter x 22 cm) on a 55°C surface, and the latency to perform one of the following nociception responses: hind paw licking, hind paw withdrawal/shaking, or jumping was timed with a 35 s maximum cutoff time to prevent tissue damage. The tail flick test was performed using an ITC Life Science Tail Flick Analgesia Meter to aim a high-intensity light beam (45% active intensity) at the tail. The latency to tail withdrawal from the beam was timed with a 10 s maximum cutoff time to prevent tissue damage. Since tail flick is more reflective behavior, the hot plate test was performed first, followed by the tail flick test. Baseline measurements for each test were recorded prior to drug injection.

During cumulative dosing antinociception, mice (*n* = 6/group) received increasing amounts of drug over the course of the study. Immediately after obtaining baseline values for both test, U-47700 was administered by subcutaneous injection and latency to nociception was measured 15 min post-injection. Testing was repeated in 15 min intervals until full antinociception was observed in both assays (i.e., maximum cutoff time was reached). U-47700 was tested at the following intervals to generate a full dose-response curve: 0.2, 0.4, 0.8, 1.2, 1.6, 2.0 mg/kg.

Antinociception data were transformed from time to percent maximum possible effect (%MPE), which is calculated as: $\%MPE=\frac{\left(test-baseline\right)}{\left(cutoff-baseline\right)}\times 100.$ (Bremer et al., 2014)

These data were then fit using a log(agonist) vs. normalized response nonlinear regression in GraphPad PRISM 8. The ED_50_ values were determined for each antinociception test and individual treatment groups, and subsequently used to determine potency ratio.

### 2.8 Blood-brain distribution

Mice (*n* = 6/group) were injected subcutaneously with 0.2 mg/kg of U-47700. Approximately, 15 min post-injection, the mice were anesthetized with isoflurane and rapidly decapitated using a sharp guillotine. The trunk blood and brain samples were surgically separated. The brain was weighed (typically 0.4–0.5 g) and added to an equal volume of PBS (1:1 ratio, 0.4–0.5 mL), in a microcentrifuge tube prefilled with zirconium oxide beads (Next Advance ZrOB05). The sample was homogenized using a bullet Blender^®^ homogenizer (Next Advance). The trunk blood was centrifuged at 13,000 rpm for 15 min to collect sera. Brain and serum samples were stored at −80°C until further analysis.

To create the standard curve, 100 μL of blank serum or brain samples were spiked with 50 μL of 50 ng/mL deuterated internal standard in acetonitrile (ACN) and 50 μL of drug at concentrations of 63, 125, 250, 500, and 1,000 ng/mL in ACN (Bremer et al., 2016). The deuterated drug that was added to the sample as an internal standard was U-47700_d_6_. The samples were mixed thoroughly through intensive vortexing. After vortexing, 100 μL of 50 mM sodium carbonate (Na_2_CO_3_) was added to adjust sample pH, and followed by 700 μL of ethyl acetate, and the sample were vigorously vortexted for the extraction. Following centrifugation at 3000 rpm for 5 min, 500 μL of the top solvent layers were removed, transferred to a new tube, and evaporated using Genevac. The resulting residues were reconstituted in 100 μL of methanol before analysis by LC-MS. The standard curve was generated by plotting the drug concentration divided by the factor of two (ratio of sample volume over spiked drug volume), which were 31, 63, 125, 250 and 500 ng/mL, against the corresponding ratio of drug/internal standard signal.

On the day of analysis, frozen brain and serum samples were thawed at room temperature, and 100 μL of serum or brain samples were spiked with 50 μL of 50 ng/mL deuterated internal standard and 50 μL of PBS. Following the same sample processing methods as previously stated, the drug concentrations in the samples were calculated by referencing the standard curve using the ratio of drug/internal standard signal. The values in the brain samples should be multiplied by two due to the 1:1 PBS dilution.

The samples were analyzed using an Agilent 6135 Single quadrupole mass spectrometer coupled with an Agilent 1260 LC stack equipped with a DAD detector. A Poroshell 120 EC-C8 column (2.1 × 100 mm, 2.7 μm) was used for chromatographic separation. The chromatographic separation was achieved using a mobile phase gradient starting from 95% H_2_O in ACN, which was ramped up to 95% ACN over a period of 10 min. This was followed by an isocratic phase of 95% ACN for 4 min at a flow rate of 0.5 mL/min. The sample injection volume was 10 μL. The mass spectrometry analysis was performed using electrospray ionization (ESI) in positive mode. Ion with mass corresponding to U-47700 (m/z 326 → 329 ESI+ and U-47700_d_6_ (m/z 334 → 335 ESI+) were extracted.

## 3 Results

### 3.1 Hapten design, synthesis, and bioconjugation

**FIGURE 2:**
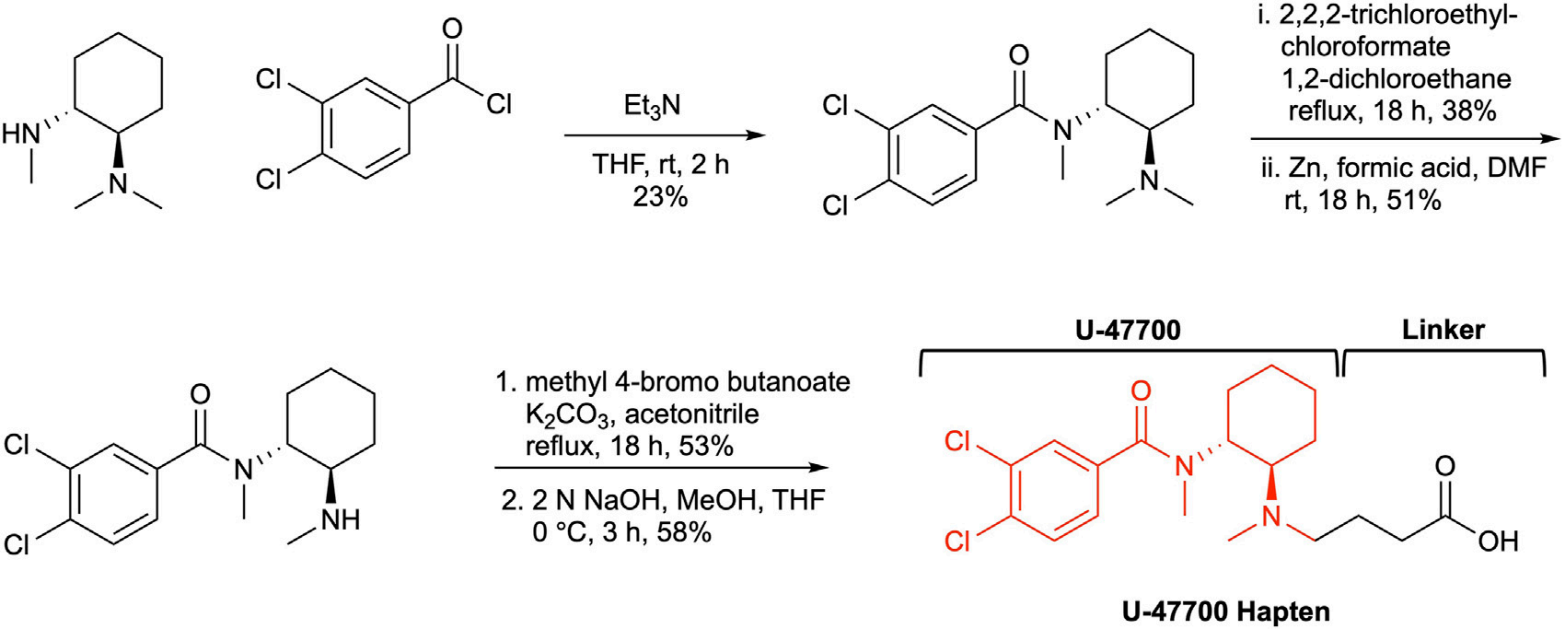
The synthesis of U-47700 Hapten.

One of the most critical aspects in drug-vaccine development is hapten design. The hapten should mirror both chemical and stereogenic characteristics of the parent drug as a reference point. Additionally, a strategically positioned linker is crucial in the hapten design, as it maintains the molecule’s structural integrity and enables easy conjugation to a carrier protein. The structure of U-47700 possesses two stereogenic centers that are embedded between two moieties, a dichlorobenzene and cyclohexyl diamine. Keeping in mind these remarks a linker would be appended within our hapten design at the *N*-methyl region on the cyclohexyl diamine (Figure 2). Following this course of action, we began our U-47700 hapten synthesis with the preparation of the original U-47700 drug, following known procedures (Szmuszkovicz and Von Voigtlander, 1982; Cheney et al., 1985; McConnell et al., 2018). We were thus led to utilize (1*R*,2*R*)-*N*,*N*,*N*′-trimethyl-1,2-diaminocyclohexane as the starting material to conserve the chirality of the anticipated hapten. Thus, coupling with (1*R*,2*R*)-*N*,*N*,*N*′-trimethyl-1,2-diaminocyclohexane and 3,4-dichlrobenzoyl chloride provided U-47700. With the drug’s synthesis secured we next examined hapten synthesis, which required *N*-demethylation; however, reported conditions using 1-chloroethylcholoroformate/MeOH condition failed to produce the desired product. As a fallback position, we optimized the *N*-demethylation reaction using previous conditions developed in our laboratory now employing the 2,2,2,-trichloroethyl chloroformate/Zinc (Cai et al., 2013). In the penultimate step linker was attached utilizing methyl 4-bromo butanoate and from here our targeted, hapten synthesis was finalized through methyl ester hydrolysis (Supplementary Data S1).

The carboxylic acid contained within U-47700 hapten was activated with NHS for conjugation to an immunogenic carrier protein. We chose KLH as an immunogenic carrier protein for this study because this protein has high immunogenicity, is relatively inexpensive and it has been used as a vaccine carrier in human clinical studies (Eggermont et al., 2013; ClinicalTrials.gov, 2023). Hapten conjugation with BSA was conducted in parallel to analyze copy numbers of hapten-protein conjugates. The copy number of immunoconjugates was 23, which was calculated by MALDI-TOF-MS (Supplementary Figure S1).

### 3.2 Vaccine administration and antibody analysis

Formulated vaccines were administered intraperitoneally to female Swiss Webster mice on weeks 0, 2, and 4. Unconjugated KLH was used for the control vaccine. All sera were collected at week 3 and 5 by retro-orbital bleeding for antibody titer and affinity tests.

**FIGURE 3:**
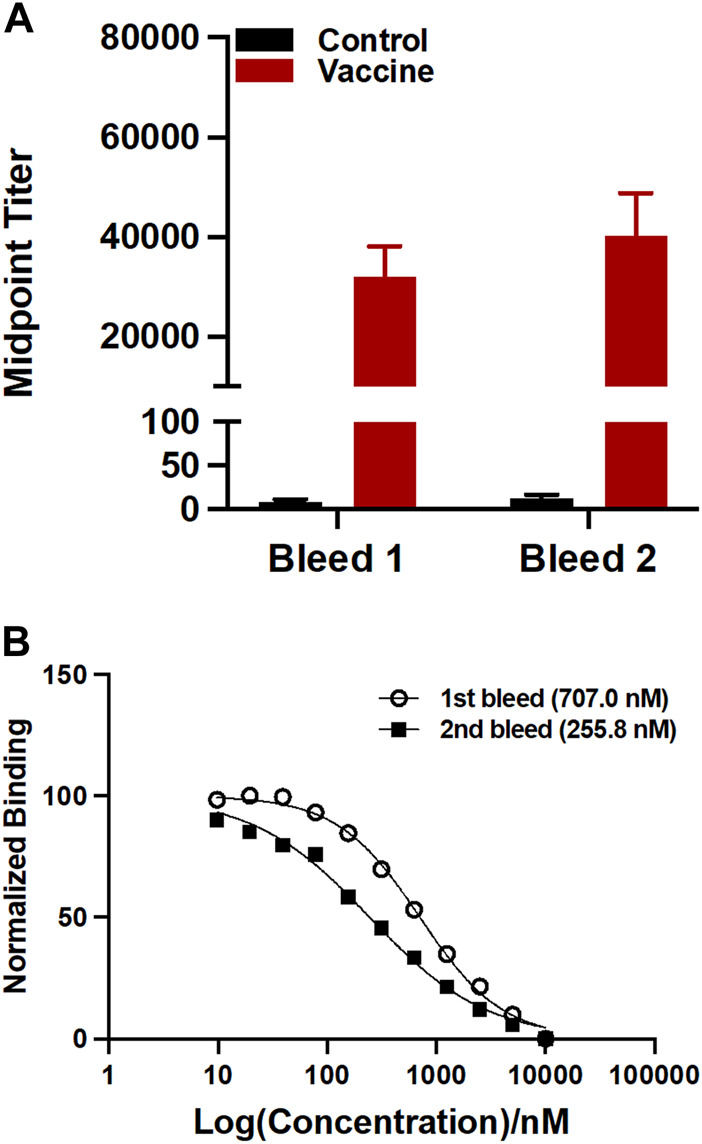
Evaluation of antibodies generated by the U-47700-KLH vaccine. **(A)** Antibody mid-point titers were measured by ELISA using serum from vaccinated mice (*n* = 6/group) on week 3 (bleed 1) and 5 (bleed 2). Bars denote means ± SEM. **(B)** Binding affinity (IC_50_) between vaccine-generated antibodies and U-47700 was determined by competitive SPR assay. Abbreviation: KLH, keyhole impact hemocyanin; ELISA, enzyme-linked immunosorbent assay; SPR, surface plasmon resonance; IC_50_, half-maximal inhibitory concentration.

The sera were analyzed by ELISA on a 96 well plate coating with U-47700-BSA conjugate to measure the antibody titers. Midpoint titers for mice vaccinated with only KLH were about 20 for the first bleed and second bleed (Figure 3A). On the other hand, the group inoculated with the drug conjugate produced titers values approximately 32,000 for the first and roughly 40,000 for the second bleed. Antibody affinities were determined through a competitive binding assay by Surface Plasmon Resonance (SPR). The vaccine produced affinities of 707.0 nM and 255.8 nM for the first and second bleed, respectively (Figure 3B).

### 3.3 Behavior studies

After completion of midpoint titers and affinities, behavior experiments were conducted to test the protective effects of the immune responses generated from the U-47700 vaccine against drug exposure. In general, opioids induce an analgesic drug effect, which reduce pain. This effect is calculated by a quantifiable time increase in nociception responses of mice upon drug exposure. However, when rodents are vaccinated with an opioid vaccine, the generated anti-drug antibodies prevent the drug from interacting with the CNS, which reduces analgesic potency of the drug (Bremer et al., 2016). In this study, hot plate and tail flick were conducted to measure nociceptive activities when the U-47700 vaccinated mice (*n* = 6) were exposed cumulatively to drug and latency was measured 15 min after drug exposure.

**FIGURE 4:**
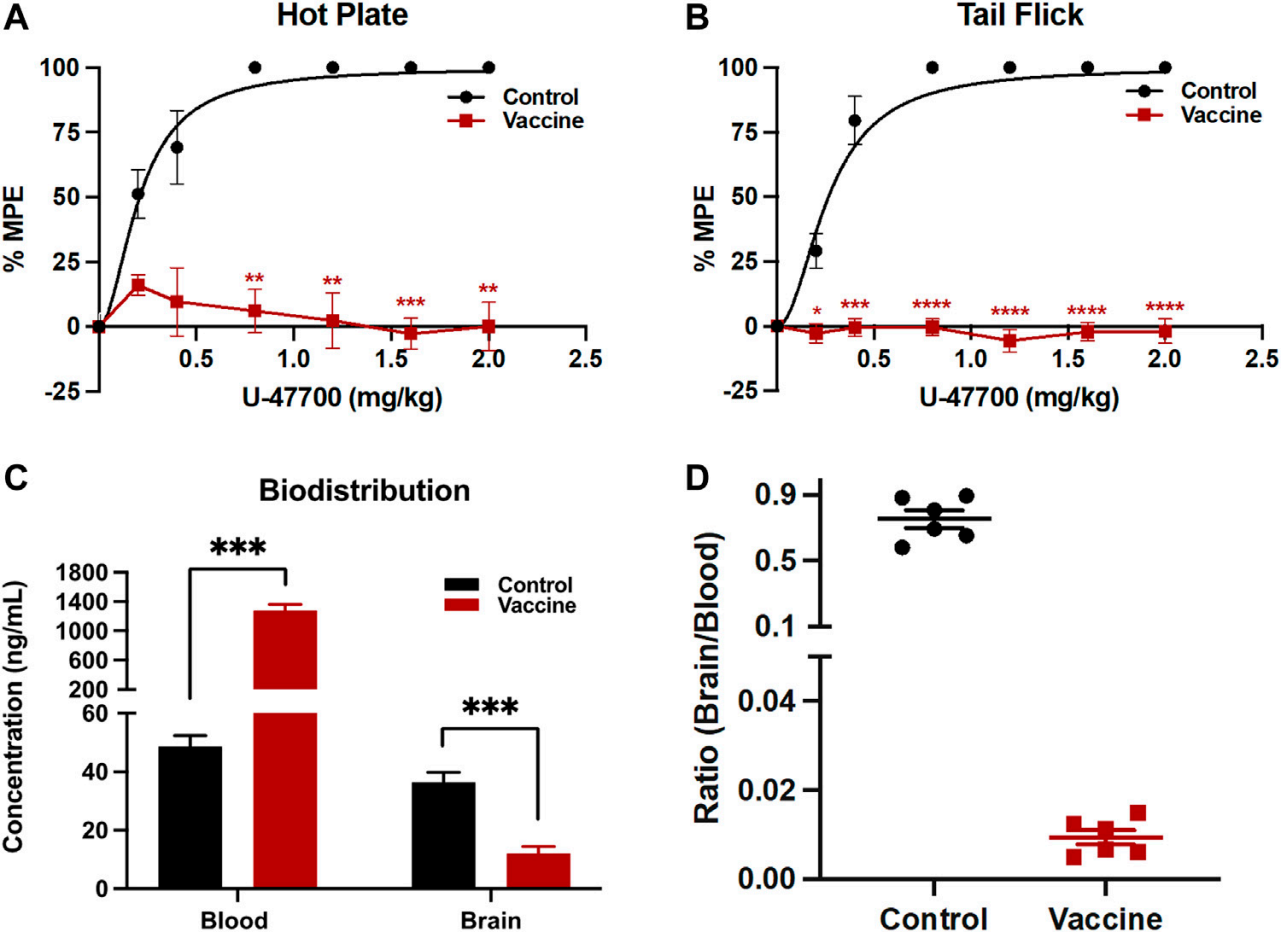
Analysis of vaccine’s protective activities by behavioral and biodistribution assays. To test antinociception activities, after baseline nociception was measured, mice cumulatively received U-47700 by subcutaneous injections and latency to nociception was measured at 15 min intervals by **(A)** hot plate and **(B)** tail flick tests. Significance is denoted by asterisks from two-way ANOVA with Bonferroni’s post hoc test to obtain the *p*-value, *p < 0.05, **p < 0.01, ***p < 0.0001, ****p < 0.00001 versus control. In the case of blood-brain biodistribution tests, mice were dosed with 0.2 mg/kg of U-47700, then whole blood and the brain were harvested 15 min post-injection. Drug concentrations in serum and brain were quantified by LC-MS **(C)**. Significance is denoted by asterisks from two-way ANOVA with Bonferroni’s post hoc test, followed by multiple *t*-test corrected using the holm-sidak method comparing the vaccine effect on blood and brain separately to obtain the *p*-value, *p < 0.05, **p < 0.01, ***p < 0.0001 versus control. Bars denote means ± SEM in all plots; *n* = 6 per group for all studies. Ratios of drug concentrations in brain over drug concentrations in serum were determined for each individual mouse **(D)**.

Vaccine efficacy can be calculated by the half dose of drug to show full antinociceptive effects on the animals. In these experiments, the ED_50_ of U-47700 in Swiss Webster mice was determined from unvaccinated control mice, which was around 0.2 mg/kg. Vaccinated mice failed to reach full antinociceptive effects even at maximum dose of 2.0 mg/kg due to the therapeutic antibody’s protective effects (Figure 4A, B).

### 3.4 Blood-brain barrier studies

To examine the ability of the U-47700-KLH vaccine to alter drug biodistribution, blood and brain samples of drugged control and vaccinated mice after their drug exposure were analyzed. A successful vaccine should sequester the drug and thus blunt brain entry by generating an antibody-drug complex in the periphery, which cannot penetrate the blood-brain barrier. In this study, unvaccinated and vaccinated mice received subcutaneously 0.2 mg/kg of U-47700 and were sacrificed 15 min later. The concentrations of U-47700 in brain and blood were measured by LC-MS. In the unvaccinated mice, the concentrations of U-47700 were 48.6 ng/mL in the blood samples and 36.4 ng/mL in the brain samples. Conversely, the mice vaccinated with U-47700-KLH displayed 1,280.9 ng/mL of U-47700 in the blood and 12.0 ng/mL in the brain. Upon analyzing the ratio of brain/blood drug concentration in individual mice, it is evident that non-vaccinated mice exhibited ratios close to 1, indicating unhindered diffusion of the drug between the brain and blood compartments. However, in vaccinated mice, the distribution pattern was significantly disrupted, resulting in a substantial retention of the drug within the bloodstream (Figure 4D).

## 4 Discussion

The rise of new synthetic opioids complicates the opioid crisis in America as these compounds often escape the traditional illicit drug classifications established by law enforcement and may be more potent and lethal than conventional molecules (Madras, 2017). A recent addition to the NPS chemotypes, U-47700, has been discovered as an adulterant in a number of abused drugs including opioids and synthetic cannabinoids (Szmuszkovicz and Kalamazoo, 1978; Szmuszkovicz and Von Voigtlander, 1982). It has been postulated that U-47700 upsurge in abuse to be tied to its ease of preparation, typically within clandestine laboratories and its potency, as stated *vide supra* approximately 10-fold greater than morphine (Kundu et al., 1999; Coopman et al., 2016; Domanski et al., 2017; Ellefsen et al., 2017). To combat its *μ*-opiate pharmacology, we formulated a U-47700 vaccine that produced an immune response with robust titers and affinities, which was central in generating compelling behavioral and biodistribution data. The grounding of our vaccine’s success was undoubtably hapten driven in that it mirrored the U-47700s key chemical epitopes. However, equally important strategy of hapten design was a linker appended at the methyl position within the cyclohexyl diamine ring, this thus allowed us to maintain a close representation of the U-47700 structure.

The antibodies generated from the U-47700 vaccine were characterized by ELISA for antibody concentration and SPR for affinity. As shown in Figure 3A, our U-47700 vaccine induced a robust antibody response throughout the study. Noted was a slight increase of the second bleed midpoint titer indicating that immune response was maintained throughout boost injection, while antibodies produced by the U-47700 vaccine possessed consistent sub-micromolar IC_50_ values against the drug. Moreover, the increase in affinity between first and second bleeds, which was 707.0 nM and 255.9 nM, respectively, suggested an enhancement of the immune response. Based on the results from titers and affinities, the conjugate vaccine was successful in generating drug specific polyclonal antibodies against U-47700.

The elicitation of antibodies with high titers and sub-micromolar affinity to U-47700 correlated well with the vaccine’s ability to modulate drug effects. Importantly, we did not observe full recovery of antinociceptive effects in vaccinated mice. Even the nociceptive latency in the vaccinated mice at a cumulative amount of 2.0 mg/kg of U-47700 (about 10-fold greater than the ED_50_ of the drug in control mice) remained at baseline levels. This noteworthy result was also observed in a previous opioid vaccine study (Eubanks et al., 2021), where mice vaccinated with Carfen-ester-*TT* did not experience drug effects when exposed to concentrations 100-fold greater than the ED_50_ of carfentanil. Therefore, because the vaccinated mouse did not reach maximum drug effects when they exposed high concentration of U-47700, we believe our U-47700 vaccine would protect against drug effects including respiratory depression. However, due to the fact that the animals were sacrificed for biodistribution studies, we could not add a plethysmography experiment in this study.

In addition to this nociceptive data, the protective effects of the U-47700-KLH vaccine were also supported by biodistribution studies. As shown in Figure 4C 26-fold difference of U-47700 was detected in the blood samples of U-47700-KLH vaccinated mice compared to unvaccinated mice. Conversely, the vaccinated group also displayed reduced concentrations of U-47700 in the brain when compared to the unvaccinated group. In sum the biodistribution data provide another piece of evidence that the generated antibodies from the U-47700 vaccine successfully blocked the drug from crossing the blood-brain barrier, which in turn explain the significant protective effects we observed in the behavioral assay.

## 5 Conclusion

U-47700 is a potent *μ*-opioid receptor agonist initially discovered by the Upjohn company (Szmuszkovicz and Kalamazoo, 1978; Szmuszkovicz and Von Voigtlander, 1982) was originally created as a substitute for morphine but unfortunately has become one of the most recent additions of clandestine synthetic opioids being used as an adulterant (Madras, 2017; Baumann et al., 2018). Like other synthetic opioids, U-47700 produces analgesia, pronounced euphoria, and sedation, ultimately leading to serious respiratory depression (Domanski et al., 2017). Since the first death in 2016, many fatalities worldwide are associated with the use of U-47700 (Alzghari et al., 2017; Schneir et al., 2017). Regrettably the only current treatment for U-47700 misuse is naloxone, thus a need for new pharmaceutics to combat U-47700 abuse and overdose. Our laboratory like many others has sought ways to address the opioid global health problem, wherein we have focused our efforts upon the development of opioid conjugate vaccines (Bremer and Janda, 2017; Olson et al., 2018; Lee et al., 2022). The design and synthesis of a U-47700 hapten and its evaluation *in vivo* revealed the successful generation of antibody titers and affinities, which produced potent protective effects upon drug exposure in behavioral and biodistribution assays. The methodologies developed herein provides the basis for further investigations to develop more innovative therapeutic models against NPS opioids, which might help to address opioid-related fatalities.

## Data Availability

The original contributions presented in the study are included in the article/Supplementary Material, further inquiries can be directed to the corresponding author.
