# Supplementary material for: Development of a vaccine against the synthetic opioid U-47700
Source: Front Pharmacol. 2023 Jul 10;14:1219985. doi: 10.3389/fphar.2023.1219985 (PMC10363602; doi:10.3389/fphar.2023.1219985)
Supplement: Supplementary file 1 [file DataSheet1.DOCX]

Supplementary Material

Development of the vaccine against novel synthetic opioid U-47700

Hyeri Park, Mingliang Lin, Jian Zhou, Lisa M. Eubanks, Bin Zhou, Kim D. Janda*

*** Correspondence:** Kim D. Janda: kdjanda@scripps.edu

# Supplementary Figures

## Supplementary Figures

 **Supplementary Figure S1.** MALDI-ToF of U-47700-BSA immunoconjugate

# Supplementary Data

## Synthesis of U-47700 hapten

**3,4-Dichloro-*N*-((1*R*,2*R*)-2-(dimethylamino)cyclohexyl)-*N*-methylbenzamide (U-47700)**: Triethylamine (0.49 mL, 3.5 mmol) was added to the solution of (1*R*,2*R*)-*N*,*N*,*N*’-trimethyl-1,2-diaminocyclohexane (500 mg, 3.2 mmol) in anhydrous THF (5 mL) under Ar atmosphere. The solution of 3,4-dichlorobenzoyl chloride (731.9 mg, 3.5 mmol) in anhydrous THF (5 mL) was added dropwise to the resulting solution at 0 ºC. On completion of the addition, the reaction mixture was stirred for 2 h at rt. The resulting mixture was diluted with EtOAc and H_2_O. The layers were separated, the aqueous layer was extracted with EtOAc. The combined organic layers were dried over Na_2_SO_4_ and concentrated *in vacuo*. The residue was purified by flash column chromatography (silica gel, CH_2_Cl_2_/MeOH, 9/1 to 4/1) to afford U-47700 (240 mg, 22.8%).

**3,4-Dichloro-*N*-methyl-*N*-((1*R*,2*R*)-2-(methylamino)cyclohexyl)benzamide**: 2,2,2-Trichloroethyl chloroformate (2.1 mL, 15.2 mmol) was added dropwise at 0 ºC to the solution of U-47700 (1g, 3.0 mmol) in 1,2-dichloroethane (15.2 mL). The reaction mixture was refluxed overnight. The resulting mixture was diluted with EtOAc and H_2_O. The layers were separated, the aqueous layer was extracted with EtOAc. The combined organic layers were dried over Na_2_SO_4_ and concentrated *in vacuo*. The residue was purified by flash column chromatography (silica gel, Hexane/EtOAc, 1/1 to 1/2) to afford the intermediate (560 mg, 37.5%). Zinc powder (447 mg, 6.8 mmol) was added to the solution of the intermediate (560 mg, 1.1 mmol) in DMF (15 mL) and, then formic acid (21 mL) was added dropwise. The mixture was stirred at rt overnight. The resulting mixture was purified by flash column chromatography (silica gel, CH_2_Cl_2_/MeOH, 4/1 to 1/1) to afford the title compound (184 mg, 50.9%).: ^1^H NMR (500 MHz, CDCl_3_) δ 7.79 (d, *J =* 1.9 Hz, 1H), 7.66 (dd, *J =* 8.3, 2.0 Hz, 1H), 7.45 (d, *J =* 8.3 Hz, 1H), 4.65 (s, 1H), 3.50 (s, 1H), 2.92 (s, 3H), 2.70 (s, 3H), 2.29 (d, *J =* 10.7 Hz, 1H), 1.93–1.79 (m, 3H), 1.75–1.59 (m, 2H), 1.54–1.15 (m, 3H).

**Methyl 4-(((1*R*,2*R*)-2-(3,4-dichloro-*N*-methylbenzamido)cyclohexyl)(methyl)amino)butanoate**: Potassium carbonate (58 mg, 0.4 mmol) was added to the solution of 3,4-dichloro-*N*-methyl-*N*-((1*R*,2*R*)-2-(methylamino)cyclohexyl)benzamide (44 mg, 0.1 mmol) in acetonitrile (1.5 mL). The mixture was stirred at rt for 1.5 h and then methyl 4-bromo butanoate (38 mg, 0.2 mmol) was added. The reaction solution was refluxed overnight. The resulting mixture was filtered, and the filtrate was evaporated under reduced pressure. The residue was purified by flash column chromatography (silica gel, Hexane/EtOAc, 7/3 to 1/1) to afford the title compound (30.6 mg, 52.9%).: ^1^H NMR (500 MHz, CDCl_3_) δ 7.50 (d, *J* = 1.9 Hz, 1H), 7.46 (d, *J* = 8.2 Hz, 1H), 7.23 (dd, *J* = 8.2, 2.0 Hz, 1H), 3.66 (d, *J* = 14.8 Hz, 3H), 2.84 (d, *J* = 72.4 Hz, 3H), 2.63 – 2.53 (m, 1H), 2.48 (td, *J* = 11.6, 3.6 Hz, 1H), 2.38 – 2.32 (m, 2H), 2.30 (d, *J* = 7.3 Hz, 1H), 2.21 (s, 2H), 1.87 (s, 2H), 1.85 – 1.78 (m, 2H), 1.74 (td, *J* = 13.8, 6.7 Hz, 3H), 1.66 (s, 1H), 1.48 – 1.38 (m, 1H), 1.32 – 1.22 (m, 1H), 1.21 – 0.94 (m, 2H); ^13^C NMR (125 MHz, CDCl_3_) δ 174.16, 168.89, 137.41, 137.11, 133.32, 132.74, 130.46, 129.11, 126.24, 64.20, 59.66, 51.51, 36.24, 35.82, 31.50, 29.51, 27.60, 25.44, 23.59, 22.83; HRMS (ESI) *m/z* 415.1533 [(M+H)^+^ calcd for C_20_H_29_Cl_2_N_2_O_3_ 415.1550].

**4-(((1*R*,2*R*)-2-(3,4-Dichloro-*N*-methylbenzamido)cyclohexyl)(methyl)amino)butanoic acid**: 2 N NaOH (4 mL) and THF (10 mL) were added to the solution of methyl 4-(((1*R*,2*R*)-2-(3,4-dichloro-*N*-methylbenzamido)cyclohexyl)(methyl)amino)butanoate (30.6 mg, 0.07 mmol) in MeOH (4 mL) at 0 ºC. The reaction mixture was stirred at 0 ºC for 3 h. The resulting mixture was acidified with the addition of saturated citric acid to adjust pH 4 and transferred to the separate funnel. The aqueous phase was extracted with EtOAc. The combined organic layers were dried over Na_2_SO_4_ and concentrated *in vacuo*. The residue was purified by reverse phase column chromatography (RediSep Rf Gold C_18_, gradient: 10–100% H_2_O/Acetonitrile in 0.1% formic acid) to afford the hapten (17 mg, 57.5%).: ^1^H NMR (500 MHz, CDCl_3_) δ .60 (s, 1H), 7.51 (d, *J* = 7.8 Hz, 2H), 2.95 (d, *J* = 43.7 Hz, 2H), 2.83 (s, 3H), 2.63 (d, *J* = 32.8 Hz, 2H), 2.50 (s, 4H), 2.05 (d, *J* = 12.2 Hz, 1H), 1.87 (d, *J* = 25.6 Hz, 6H), 1.61 (s, 1H), 1.42 (d, *J* = 13.2 Hz, 2H), 1.29 – 1.19 (m, 1H); ^13^C NMR (125 MHz, CDCl_3_) δ 175.82, 170.41, 136.38, 133.72, 132.58, 130.65, 129.26, 126.84, 64.01, 52.67, 36.32, 31.77, 31.72, 29.91, 24.96, 24.76, 23.17, 20.90; HRMS (ESI) *m/z* 401.1385 [(M+H)^+^ calcd for C_19_H_27_Cl_2_N_2_O_3_ 401.1393].

**
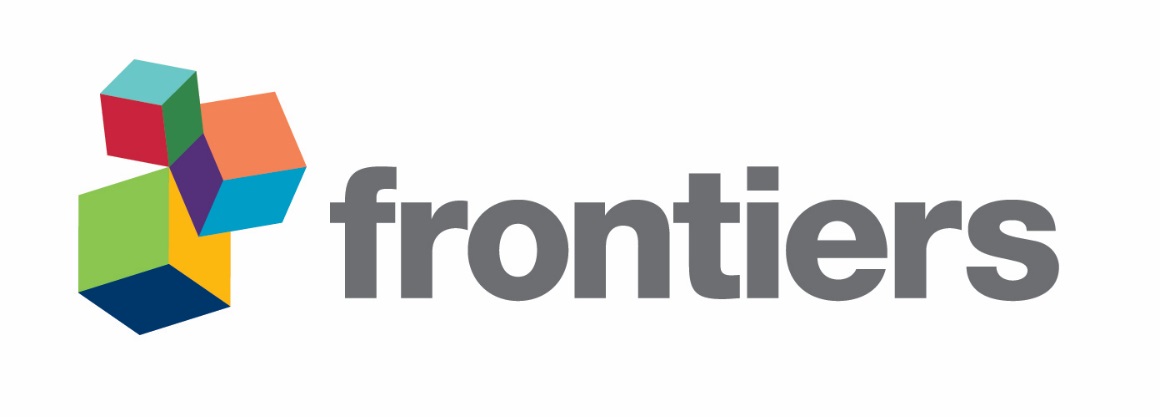
**
